# Temperature influences mood: evidence from 11 years of Baidu index data in Chinese provincial capitals

**DOI:** 10.3389/fpubh.2025.1569903

**Published:** 2025-05-15

**Authors:** Mengjiao Yin, Mengmeng Zhu

**Affiliations:** School of Business, Wuxi Taihu University, Wuxi, China

**Keywords:** temperature effects, environmental psychology, negative moods, Baidu index, mental health

## Abstract

**Introduction:**

This study explores the dynamic relationship between temperature changes and public negative emotions—specifically depression, anxiety, and loneliness. It introduces an innovative approach by integrating climate data with digital behavior metrics to provide objective insights into how environmental factors may influence mental health.

**Methods:**

A dataset combining daily meteorological records and Baidu search indices from 31 provincial capital cities in China (2013–2023) was used. Search engine query data served as a proxy for public emotional states, avoiding social desirability bias commonly found in self-reported surveys. Panel fixed-effect models and heterogeneity analysis were employed to assess the impact of daily mean temperature (DMT) and daily temperature range (DTR). A “climate zone × season” framework was constructed to explore regional and seasonal variations. Threshold regression analysis was also conducted to identify nonlinear effects.

**Results:**

The results showed that for every 1°C increase in DMT, search indices for depression, anxiety, and loneliness increased significantly by 22.71%, 18.76%, and 19.59%, respectively (*p* < 0.01). Conversely, a 1°C increase in DTR led to decreases of 30.35%, 31.19%, and 15.41% in these indices (*p* < 0.05). Threshold regression analysis revealed that the adverse effect of high temperatures on loneliness became insignificant when DTR exceeded 14°C. Heterogeneity analysis highlighted significant regional and seasonal differences, particularly during cold seasons in severely cold zones and hot seasons in warm summer-cold winter zones.

**Discussion:**

The findings suggest that temperature fluctuations have a complex and regionally dependent impact on public mental health. The moderating role of climate characteristics and seasonal patterns underscores the importance of localized climate policies and mental health interventions. This study provides empirical evidence based on objective behavioral data, contributing to climate-related public health strategies and adaptive policy design.

## 1 Introduction

**Figure 1:**
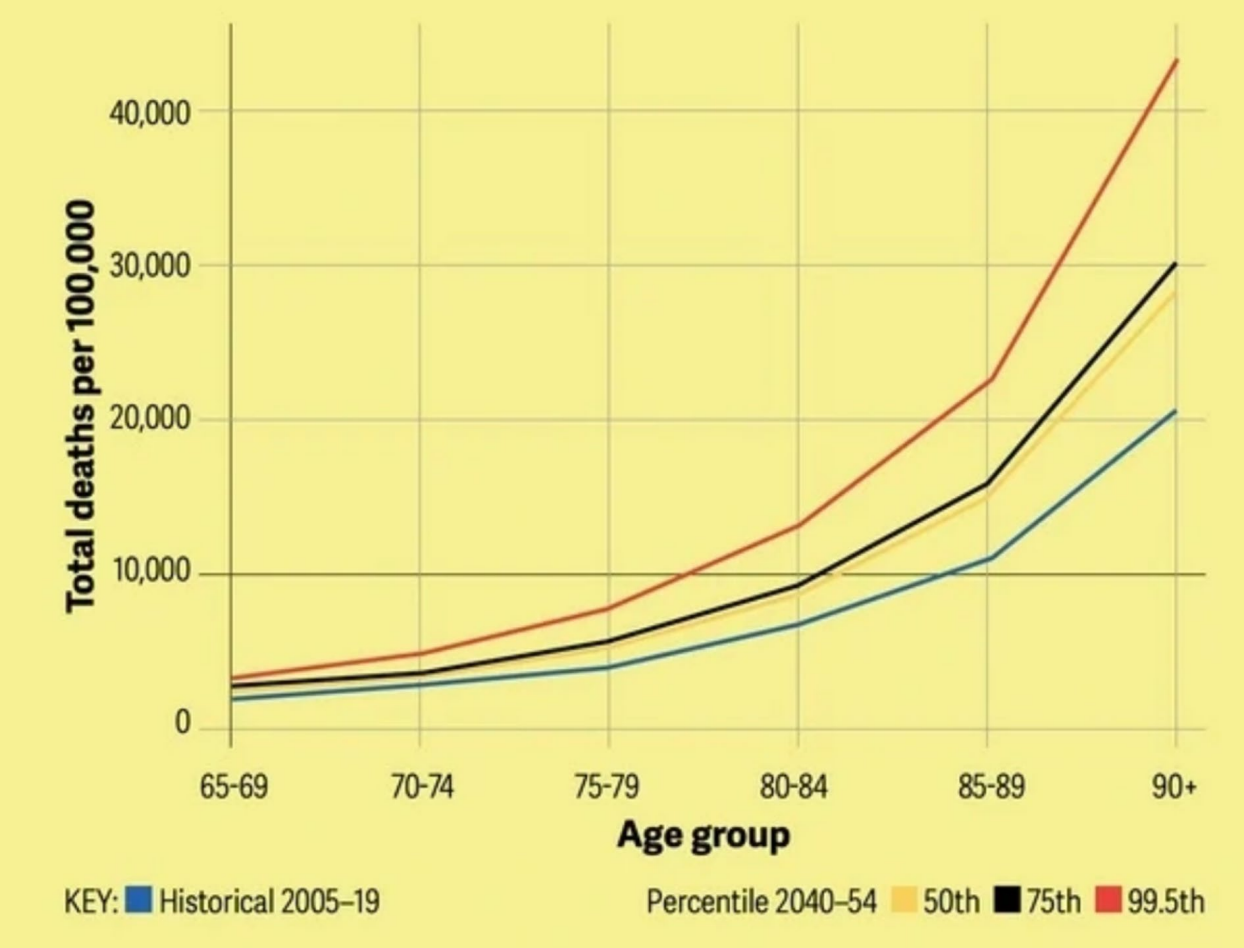
Predicted mortality due to heat events [Source: Report titled “Boiling Point” by Gill et al. (2)].

The negative impacts of global climate change on public health cannot be underestimated. Extreme heat waves are one of the most direct effects of climate change on human health. Liu et al. (1) found that 14% of resident deaths are related to uncomfortable environmental temperatures (too high or too low), and the long-term impact of cold temperatures on mortality risk (lasting more than 2 weeks) is stronger than that of heat (usually lasting 2–3 days). Gill et al. (2) explored the potential impacts of global warming on human health and mortality in their study, presenting predicted mortality rates for different temperature percentiles across age groups between 2040 and 2054 (see Figure 1). The predictions show that compared to historical data from 2005 to 2019, future extreme heat events will significantly increase the risk of death. According to the IPCC (60) report, in 2019, deaths from climate-sensitive diseases globally amounted to 39,503,684, accounting for 69.9% of total deaths that year (3). Cardiovascular disease was the leading cause, representing 32.8%. Without action to limit global warming, 3.4 million people could die annually from climate-related illnesses by the end of this century (4).

Mental health is also a critical component of public health, and studies have shown that negative emotions are affected by temperature. A survey of 1.9 million people indicated that when temperatures exceed 21°C, positive emotions (such as happiness and joy) significantly decrease while negative emotions like anger and fatigue increase, with higher temperatures having a stronger effect (5). The analysis of social media sentiments also shows that rising temperatures exacerbate negative emotions. For instance, an analysis of 80 million tweets in Argentina revealed that high temperatures lead to an increase in negative emotions (such as the frequency of vulgar language), which could potentially trigger real-world violent behavior. This calls for early warning systems and public health interventions (6). Similarly, an examination of 400 million posts on Weibo in China showed that extreme heat is one of the primary drivers of negative emotional expression on social media, and climate change could worsen daily emotional expression by 0.3–2.1% (7). A review article mentioned that high temperatures are closely associated with increased incidences of depression, mania, bipolar disorder, etc. Short-term exposure to high temperatures can trigger depressive symptoms, whereas long-term exposure significantly increases the risk of major depression. During heatwaves, hospitalization rates for cerebrovascular diseases and schizophrenia patients also notably rise (8).

Despite a substantial body of literature examining the relationship between temperature and mood, several limitations remain to be addressed. Firstly, most studies focus on Western countries or small-sample experimental settings, with empirical analyses particularly lacking for developing countries like China—a giant economy notable for its climatic diversity and unique socio-cultural context. Secondly, existing research often relies on short-term experimental data under laboratory-controlled conditions or individual self-report data (such as surveys and diaries), which struggle to capture long-term emotional fluctuation patterns among large populations in real-life scenarios. Moreover, while some studies have utilized digital behavioral trace data, such as He et al. (9) using online review data from Chinese restaurants and Molina et al. (10) analyzing text data from Spanish Twitter users, no study has systematically integrated climate data with search behavior to uncover the dynamic association mechanism between temperature changes and public mood. According to the above limitations, the objective of this study is to deeply explore the relationship between different aspects of temperature (mean temperature reflecting central tendency, temperature range reflecting fluctuation tendency) and search behavior.

This study’s innovation is reflected in three aspects: Firstly, by leveraging Baidu Index data spanning 11 years (2013–2023) across provincial capitals in China, this research transcends traditional geographical limitations and sample size constraints, marking the first attempt to combine climate big data with internet behavior data to reveal the macro impact mechanism of temperature changes on public mood. Secondly, the study focuses on “regional-seasonal” differences, employing a sophisticated framework encompassing five climatic zones (severe cold, cold, hot summer-cold winter, hot summer-warm winter, and temperate) and three temperature conditions (summer, winter, and transitional seasons) to comprehensively explore heterogeneity. Lastly, the study innovatively uses “mood-related search term popularity” as an indicator of mood, which, compared to individual subjective reports, more easily avoids social desirability bias and can reflect the spatial–temporal distribution characteristics of group mood states in real-time. These innovations enable this study to more comprehensively analyze the complex relationship between temperature and mood, providing empirical evidence with both theoretical value and practical significance for climate policy and mental health interventions.

## 2 Literature review

Researchers have been concerned with the impact of weather on mood since the 20th century. Early studies such as Sanders and Brizzolara (11) used questionnaire methods to analyze the relationship between weather indicators like temperature, air pressure, and precipitation and human emotions but did not find significant effects, a conclusion at odds with common sense that prompted subsequent scholars to continuously verify [e.g. (12)]. Although Huibers et al. supported this view through large-scale depression screening data, more research challenged this conclusion. For instance, Denissen et al. (13) found that temperature, wind force, and sunshine significantly affect negative moods using online diaries; Spasova (14) diary study showed that bad weather (such as cloudy or rainy days) increases negative emotions, while sunny weather boosts positive emotions; Ettema et al. (15) discovered through questionnaires that weather has a significant effect on travelers’ moods and satisfaction. Keller et al. (16) further indicated that the influence of weather on emotions and cognition may be moderated by seasons and outdoor time: pleasant weather (such as higher temperatures or pressures) in spring enhances mood and “cognitive breadth” with increased outdoor time, whereas under high summer temperatures, the same weather conditions can decrease mood. This finding partially explains the contradictory conclusions of early studies and emphasizes the potential impact of seasonal differences on results. Additionally, Lee et al. (17) cross-cultural study (including Japanese bank employees and American online workers) showed that bad weather might increase individual productivity by reducing cognitive distractions (such as the temptation of outdoor activities), providing a new behavioral perspective on the relationship between weather and mood. Messner and Vänke (18) conducted a diary study in Switzerland showing that self-awareness could weaken the recognition of good weather among those with positive moods. This study revealed how internal factors influence our perception of weather and its emotional changes, enriching the understanding of the complex relationship between weather and mood.

When exploring the direct impact of temperature on mood, early studies had already revealed key clues. Bell and Baron (19) experimentally found that high temperatures trigger aggressive behavior by increasing negative emotions, and Howarth and Hoffman (20) pointed out that high temperature and humidity impair attention and amplify negative experiences. This line of research extended to He et al. path analysis in China, which found that temperature not only enhances the sentiment of online reviewers but also mediates between temperature and ratings. More recent studies such as Hua et al. (21) panel analysis emphasized that temperatures exceeding thresholds significantly increase the risk of depression, especially for middle-aged, older adult, and female populations. Behnke et al. (22) showed that participants’ moods slightly improved in cooler environments, but the effect was weak. Bella Hamilton (23) conducted research in extreme and moderate temperatures in Alaska, Arizona, and Florida, discovering that when temperatures fell below −40°F or rose above 110°F, participants’ mood scores significantly decreased (1/5), whereas temperatures of 70–80°F enhanced them to 5/5. These results complemented Keller et al. findings on the beneficial effects of pleasant spring weather and Lee et al. productivity enhancement mechanism, further validating the moderating role of temperature thresholds on mood.

Simultaneously, Fischer et al. (24) conducted a systematic review exploring the role of thermosensation and thermoregulation in anxiety disorders, providing foundational insights into how temperature influences emotions through physiological mechanisms. The social thermoregulation theory posits that lower environmental temperatures may stimulate the need for social connections, while higher temperatures can promote feelings of closeness and social behaviors (25). Research by Inagaki and Human (26) also shows an association between changes in core body temperature and perceived social connection, indicating that warm environments might enhance positive emotions and social experiences. Lynott et al. (27) comprehensive analysis on the effects of temperature on prosocial and antisocial behavior, although failing to confirm the hypothesis that warmth promotes prosocial behavior, points directions for future research. These studies collectively underscore the profound impact of temperature on human emotions and social behavior.

Temperature fluctuations significantly affect cognitive and emotional functions. Falla et al. (28) systematic review indicates that cold exposure impairs attention, processing speed, memory, and executive function in healthy adults, but does not affect reasoning ability, suggesting selective impacts of low temperatures on mood and cognition. Conversely, Gaoua et al. (29) found that under passive hyperthermic conditions, working memory resources diminish, particularly during complex tasks, further demonstrating the dual negative impact of extreme temperatures on emotion and cognition. Hancock et al. (30) meta-analysis also shows that temperatures outside the comfort range (above 25.7°C or below 15°C) significantly impair cognitive performance, which could indirectly lead to changes in emotional states. Overall, both low and high extremes of temperature challenge human cognitive function and emotional stability, emphasizing the importance of maintaining suitable temperatures to ensure optimal cognitive performance and emotional health.

When temperatures reach extreme levels, they can have significant effects on mental health. For example, Burke et al. (31) noted that above-average temperatures are significantly associated with interpersonal and intergroup conflicts, suggesting that high temperatures may exacerbate negative emotions and aggressive behaviors. Thompson et al. (32) further discovered that extreme heat (such as daily maximum temperatures exceeding 35°C or prolonged heatwaves) increases the risk of hospitalization for mental illnesses and is linked to rising suicide rates, highlighting the specific threats posed by high temperatures to mental health. Li et al. (33) comprehensive analysis revealed the impact of climatic exposure on mental and behavioral health, particularly noting that both extreme cold and heat challenge the human thermoregulatory system and cause psychological stress, underscoring the broad impact of temperature extremes on emotional and behavioral health. Additionally, Ryti et al. (34) extensively discussed the relationship between cold spells and adverse health effects, including chronic disease mortality and mental health issues, adding to our understanding of the impact of extreme weather conditions on overall health.

In addition to temperature as a core factor, other meteorological conditions are also complexly related to mood. For example, Huibers et al. (12) and Kööts et al. (35) epidemiological and panel analyses suggested that low temperatures and reduced sunlight are associated with depressive moods, though with limited explanatory power. Molina et al. analyzed Spanish Twitter data, proposing that social media expressions of mood correlate with weather conditions, offering a new method for monitoring public mood. Baylis et al. (36) pioneering study utilized over 3.5 billion social media posts (from Facebook and Twitter), finding that low and high temperatures, precipitation, humidity, and cloudiness were all significantly associated with negative emotion expression, even after excluding weather-related posts, highlighting the pervasive impact of meteorological conditions on mood. Spasova diary study further indicated that sudden weather changes (like clouds or bad weather) trigger negative emotions, while Ettema et al. found that wind intensity exacerbates anxiety, and sunshine regulates cyclists’ moods. Furthermore, Khanthavai (37) Thai panel study showed that weather-induced moods significantly impact investment returns (SET index data), while Venz and Pundt (38) diary study in Germany found that morning weather correlates with job satisfaction but not with negative emotions, suggesting the importance of individual sensitivity differences.

On the level of individual differences and the long-term impacts of climate change, research has further revealed the complexity of population responses. For example, Bassi et al. (39) demonstrated through an American experiment that sunshine and good weather promote risk-taking behavior, with mood being a key mediating variable; Wullenkord et al. (40) showed in a German cross-sectional survey that climate anxiety positively correlates with depression and avoidance behaviors but negatively with self-protection strategies. Mertens (41) pointed out in their doctoral dissertation that outdoor activities in non-Michigan regions (with significant climatic differences like Alaska and Arizona) significantly improve mood, echoing the high mood scores of the Florida control group under suitable temperatures, emphasizing the interaction between regional adaptability and outdoor exposure. Fischer et al. (42) in a multi-country systematic review indicated that high temperatures correlate with interpersonal conflicts and rising suicide rates, whereas heavy rain is not a risk factor for mental disorders. Kraft et al. (43) experimental study further supports the informed bias theory, indicating that weather anxiety leads to inflexible responses to threats.

When discussing the impact of weather on public mood and behavior, the influence of extreme weather events on public emotions and decision-making cannot be overlooked. Ash et al. (44) demonstrated that storm-based warning systems not only affect people’s perception of tornado threats but also indirectly regulate emotional responses and evacuation behaviors by enhancing threat awareness. Similarly, Casteel (45) further revealed how tornado alert messages influence the public’s emotional state and decision-making process through clarity and timeliness of information, indicating that the way weather information is presented plays a crucial role in emotion management. Meanwhile, Coleman et al. (46) provided significant insights into the psychological mechanisms and intervention strategies for weather anxiety through the application of cognitive behavioral therapy to address severe-weather phobia, emphasizing the central role of emotion regulation in coping with weather-related stress. Additionally, Chen et al. (47) explored the formation mechanism of anxiety states from the perspective of interpretation bias, contributing to an understanding of why some individuals exhibit excessive worry or discomfort toward weather threats. This finding helps deepen our comprehension of the relationship between weather and emotion.

To sum up, from early studies focusing on the impact of single meteorological factors such as temperature on negative emotions to later explorations considering more diversified weather conditions and their effects on the mental health of different populations, scholars have gradually built a more comprehensive understanding framework, enriching our knowledge on the complex relationship between weather and negative emotions. Table 1 summarizes the selected representative literature on the research field of “weather impacts mood” in chronological order of publication.

**Table 1:** Overview of weather-mood correlation studies.

Author(s) (Year)	Country	Research method	Key findings	Data Used
Bell & Baron (19)	USA	Experimental study	High temperatures exacerbate negative emotions leading to aggressive behavior	Participant data from experiments
Howarth & Hoffman (20)	UK	Experiment and questionnaire survey	High temperature and humidity harm attention and mood, thermal discomfort amplifies negative experiences	Participant data from experiments
Huibers et al. (12)	Netherlands	Epidemiological survey	Low temperature and low daylight correlate with depressive moods, but the explanatory power is limited	Epidemiological survey data
Kööts et al. (35)	Estonia	Panel analysis	Sunlight modulates emotional intensity, weather explains a small variance	EMA data
Messner & Wänke (18)	Switzerland	Diary study	Those in positive moods rate weather more favorably, self-awareness diminishes this effect	Diary data
Spasova (14)	Bulgaria	Diary study	Sudden weather changes (cloudy/unfavorable) trigger negative emotions	Diary data
Bassi et al. (39)	USA	Price experiment	Sunshine/good weather promotes risk-taking behaviors, emotion acts as a mediator	Participant data from experiments
Ettema et al. (15)	Sweden	Panel analysis	Temperature boosts mood, wind exacerbates anxiety, sunlight affects cyclists’ negative moods	EMA data
Khanthavit (37)	Thailand	Panel analysis	Weather-induced moods significantly affect investment returns	SET index data
He et al. (9)	China	Path analysis	Temperature increases happiness, emotion mediates the relationship between temperature and ratings	Online review data
Wullenkord et al. (40)	Germany	Cross-sectional survey	Climate anxiety positively correlates with depression, avoidance behavior; negatively correlates with self-protection strategies	Cross-sectional survey data
Molina et al. (10)	Spain	Time series analysis	Social media sentiment correlates with weather	Social media data
Behnke et al. (22)	Poland	Path analysis	Cooler environments slightly boost mood, though the effect is weak	Self-reported data from individuals
Venz & Pundt (38)	Germany	Diary study	Morning weather impacts job satisfaction and burnout, through emotional regulation	Diary data
Hua et al. (21)	China	Panel analysis	High temperatures increase mortality risk, particularly harmful to specific groups’ mental health	Individual longitudinal tracking data
Kraft et al. (43)	USA	Experimental study	Weather anxiety leads to inflexible responses to threats, supporting the informed bias theory	Participant data from experiments
Fischer et al. (42)	Multiple countries	Systematic review	High temperatures correlate with conflict and suicide rates, heavy rain not a risk factor for mental disorders	Literature database

## 3 Methods

### 3.1 Study design

The overall design steps of this study are as follows: Firstly, the authors construct a temperature variable that includes daily mean temperatures (DMT) and daily temperature range (DTR) as independent variables. Subsequently, representative keywords indicative of negative emotions (such as depression, anxiety, loneliness) search indices on Baidu are selected as dependent variables. Panel data is analyzed using fixed effects models and random effects regression analysis, followed by a Hausman test to determine which effect model is more suitable. According to the recommended model from the Hausman test, the baseline model’s reliability is further validated by re-estimating with robust standard errors (VCE). If the impact of independent variables becomes insignificant under robustness estimation, threshold regression is employed to explore nonlinear effects.

**Figure 2:**
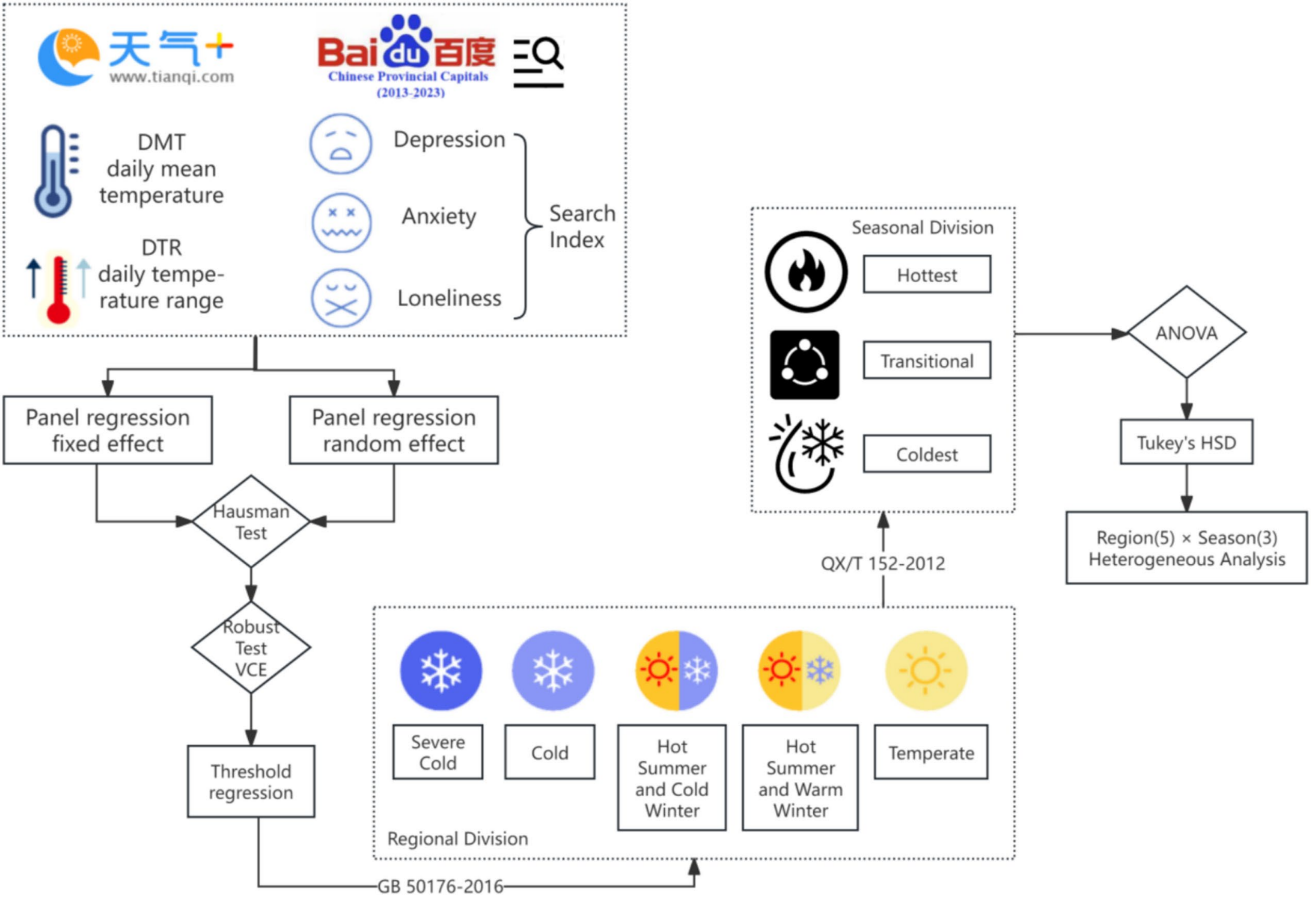
Study design.

Following this, the authors will conduct in-depth heterogeneity analysis. Based on the “Thermal Design Code for Civil Buildings” GB50176-2016, which aims to guide thermal design of buildings in different climatic zones to ensure indoor environmental comfort and energy efficiency, all case cities are categorized into five distinct climatic zones: Severe Cold Region, Cold Region, Hot Summer and Cold Winter Region, Hot Summer and Warm Winter Region, and Temperate Region. Then, according to the “Classification and Coding of Meteorological Data Part 2: Surface Observation Data” QX/T 152–2012, considering seasonal characteristics within each climatic zone, these are further divided into three types of seasonal periods: Coldest Season, Transitional Season, and Hottest Season. On this basis, inter-group variance analysis is conducted for these three seasonal periods, and Tukey’s HSD (Honestly Significant Difference) test is used for pairwise comparisons to identify differences among the three seasonal periods within different climatic zones. Finally, a cross-heterogeneity analysis of “climatic zone-seasonal period (5 × 3)” is carried out to comprehensively understand the patterns and extent of how negative emotions are influenced by temperatures. Figure 2 outlines the overall research design concept.

### 3.2 Data sources and processing

#### 3.2.1 Independent variable

Stephens-Davidowitz (48) in his book “Everybody Lies: Big Data, New Data, and What the Internet Can Tell Us About Who We Really Are” uncovers a thought-provoking phenomenon: “People lie to others but confide the truth to search engines.” He leverages Google Trends to reveal people’s genuine psychological states. The book mentions that secrets which individuals do not disclose to therapists, partners, or friends—even when symptoms are not severe enough to warrant professional help—are honestly queried to search engines, such as searching whether one truly suffers from depression. Inspired by this work, our study similarly employs a “keyword search engine popularity index,” utilizing Baidu Index to explore people’s real emotions. In China, Baidu plays a role analogous to Google, thus making Baidu Index the chosen indicator for public sentiment.

Although the authors acknowledge that these data are not as rigorously precise as medical tests, they do shed light on another aspect of daily life: natural emotional expression that is neither serious enough to require hospital visits nor constrained within laboratory settings for small-scale controlled experiments.

The dependent variables in this study come from the Baidu Index,^1^ encompassing search indices for keywords like “anxiety,” “depression,” and “loneliness.” According to official descriptions, Baidu Index analyzes keyword search trends based on vast data resources from Baidu, delving into data features such as public opinion information, market demand, and user profiles. The index updates daily, providing PC search index data since June 2006 and mobile search index data since January 2011. It reflects active search demands of netizens, where any factor affecting user search behavior could impact the Baidu Index. To ensure data authenticity, Baidu implements comprehensive anti-cheating measures, establishing a robust anti-cheating technology system to minimize the influence of fraudulent activities. Although the exact calculation method remains undisclosed due to commercial confidentiality, the authors understand that the Baidu Index is a weighted value derived from aggregated user search behavior data, with its anti-cheating mechanisms ensuring high data reliability.

**Figure 3:**
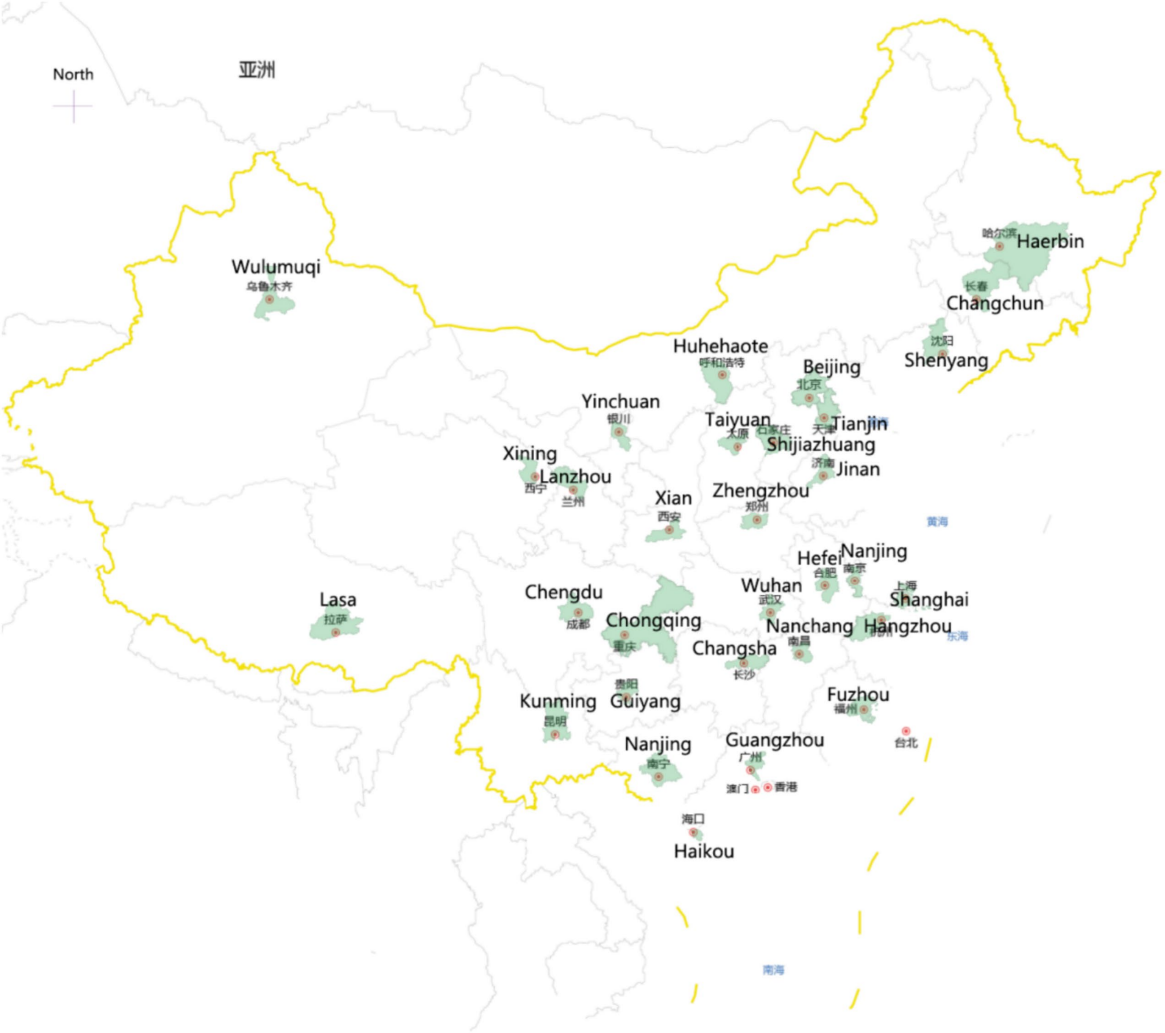
Case cities (screenshot from Geoda).

Using the open API provided by Baidu Index, the authors collected Baidu Index data for the keywords “anxiety,” “depression,” and “loneliness” from January 1, 2013, to December 31, 2023, spanning a decade, through Python programming. The collected data covers 31 provincial capital cities in China (excluding Hong Kong, Macau, and Taiwan regions where Baidu is not the primary search engine), with each province represented by its provincial capital city. Figure 3 illustrates the geographical distribution of the sample cities (marked in green) from GeoDa, a software tool designed for spatial data analysis and statistical exploration.

During the data cleaning process, to ensure the accuracy of the regression analysis, the authors removed days with missing temperature values for certain cities. Subsequently, the authors aggregated data from both PC and mobile devices to construct the final sample dataset. The sample size (N) for anxiety (anx), depression (dep), and loneliness (lon) consisted of 123,987 observations each. Specifically, the mean value for anxiety (M_anx) was 107.61 with a standard deviation (SD_anx) of 55.12, a minimum value (Min_anx) of 0, and a maximum value (Max_anx) of 538; the mean value for depression (M_dep) was 108.46 with a standard deviation (SD_dep) of 55.01, a minimum value (Min_dep) of 0, and a maximum value (Max_dep) of 1,011; the mean value for loneliness (M_lon) was 150.11 with a standard deviation (SD_lon) of 73.55, a minimum value (Min_lon) of 0, and a maximum value (Max_lon) of 787. Table 2 presents the descriptive statistics for these three dependent variables (anxiety, depression, and loneliness).

**Table 2:** Descriptive statistics of dependent variables.

Variable	Obs	Mean	Std. dev.	Min	Max
anxiety	123,987	107.6073	55.11674	0	538
depression	123,987	108.4566	55.00948	0	1,011
loneliness	123,987	150.1126	73.55175	0	787

**Figure 4:**
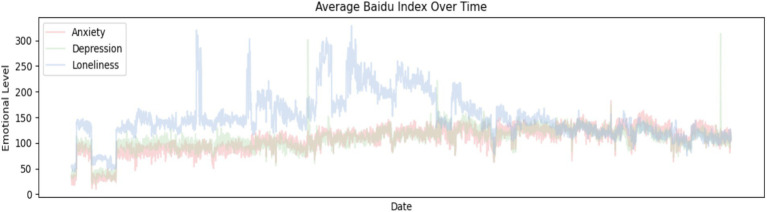
Trends in Baidu Index changes.

Figure 4 illustrates how the Baidu Index values of the keywords “anxiety,” “depression,” and “loneliness” have changed over time. This is presented in a chart, reflecting the trends in search frequency and public attention for these three keywords at different points in time.

As can be observed from Figure 4, the Baidu Index values of the keywords “anxiety,” “depression,” and “loneliness” exhibit a certain degree of correlation. Table 2 quantifies these correlations, showing a high correlation between anxiety and depression with a Pearson correlation coefficient of 0.737 (*p* < 0.001); a moderate correlation between anxiety and loneliness with a Pearson correlation coefficient of 0.524 (*p* < 0.001); and similarly, a moderate correlation exists between depression and loneliness with a Pearson correlation coefficient of 0.522 (*p* < 0.001). Therefore, statistically significant correlations are present among these three dependent variables (Table 3).

**Table 3:** Correlation of dependent variables.

Variable	Anxiety	Depression	Loneliness
anxiety	1		
depression	0.7374***	1	
loneliness	0.5236***	0.5218***	1

It is crucial to emphasize the theoretical justification for choosing the Baidu Index as the sole data source. In the early stages of research design, considering the rise of social media platforms where an increasing number of people express their sentiments, incorporating social media data was considered. However, for several reasons, the authors ultimately decided not to include social media data:

Firstly, there is a significant difference in data availability. This study’s sample period began in 2013, but many of today’s popular social media platforms were not widely used at that time, making it difficult to align their data with the timeframe provided by the Baidu Index. Secondly, algorithms for calculating popularity indices vary across platforms. Each platform uses different scales for its popularity index, which cannot be directly aggregated. Moreover, calculation methods are not open-source, making it challenging to ensure the accuracy of any weighted sum. Thirdly, there are differences in data sources. Social media popularity indices often combine post counts with search volumes, yet including post counts may compromise data reliability. For instance, news about celebrities committing suicide due to depression might be excessively hyped by marketing accounts. In contrast, the Baidu Index focuses solely on user search volume, offering a cleaner and more reliable measure. Fourthly, the Baidu Index has specific rating characteristics. As shown in the trend changes in Figure 4, despite the rise of social media, the Baidu Index does not show a declining trend, indicating it employs an internal rating algorithm to maintain consistency. Fifthly, the particular nature of the research question must be taken into account. This study examines the relationship between temperature and mood. Even if fewer users are searching on Baidu, it represents a common change in the dependent variable and does not affect correlation tests with independent variables.

#### 3.2.2 Dependent variable

Stephens-Davidowitz discovered that Americans suffer from “winter depression,” hypothesizing that moving from the cold climate of Chicago to the warm climate of Honolulu would have twice the effect on reducing negative emotions compared to antidepressant medications! This astonishing finding was the initial impetus for this study, aiming to verify if such effects also exist in China, thereby locking the independent variable as temperature. The data source is the historical weather channel of the China Weather Network (lishi.tianqi.com), which provides historical weather forecast inquiries for 2,290 regions across 34 provinces and municipalities in China. Data originates from daily weather information of cities, including historical temperatures, wind directions, wind speeds, and other historical weather conditions. This study utilized Python to scrape the highest (tmp_h) and lowest (tmp_l) temperature data from January 1, 2013, to December 31, 2023, for 31 provincial capital cities in China (excluding Hong Kong, Macao, and Taiwan, where Baidu search engine usage is not common).

During the data cleaning process, to ensure data quality, days with missing temperature values for certain cities were removed. To further guarantee data quality, the winsor2 command in Stata was applied with default parameters to process the data. This command replaces values in tmp_h and tmp_l lower than the 1st percentile with the 1st percentile value and those higher than the 99th percentile with the 99th percentile value. This measure aims to minimize the impact of extreme values caused by data recording errors on the accuracy of regression results while retaining as much data as possible.

Table 4 shows descriptive statistics of the processed independent variables, all units being Celsius. In terms of sample size, there are 123,987 observations each for daily highest temperature (tmp_h) and daily lowest temperature (tmp_l). Mean_h (mean maximum temperature) is 15.01, SD_h (standard deviation of maximum temperature) is 10.94, Min_h (minimum maximum temperature) is −15, Max_h (maximum maximum temperature) is 32.5; Mean_l (mean minimum temperature) is 9.22, SD_l (standard deviation of minimum temperature) is 3.82, Min_l (minimum minimum temperature) is 0, Max_l (maximum minimum temperature) is 32.

**Table 4:** Temperature records (After winsorization).

Variable	Obs	Mean	Std. dev.	Min	Max
tmp_h	123,987	15.01208	10.93858	−15	32.5
tmp_l	123,987	9.219015	3.821875	0	32

Subsequently, two indicators were generated as the final independent variables. The first one is DMT, representing the average temperature within a day and can be calculated by averaging the highest and lowest temperatures of the day. Its calculation formula is $\text{DMT}=\frac{\left({\text{tmp}}_{h}+{\text{tmp}}_{l}\right)}{2}$; the second one is DTR, indicating the magnitude of temperature variation within a day, which is computed by subtracting the minimum temperature from the maximum temperature. Its calculation formula is $\text{DTR}={\text{tmp}}_{h}-{\text{tmp}}_{l}$. These two metrics are used to reflect the daily average temperature level and temperature fluctuation, providing critical quantitative evidence for studying the relationship between temperature and mood.

It should be acknowledged that this method of calculating DMT is a simplified approximation approach, implying a cyclically symmetric assumption where the temperature change within a day is evenly distributed. A more refined calculation of DMT usually relies on temperature recordings at multiple points during the day to more accurately represent the actual daily temperature curve. However, due to limitations in data acquisition in this study, a simplified algorithm had to be adopted. For researchers wishing to delve deeper into this issue, it is recommended to refer to Stevenson et al., who, when dealing with similar problems, employed more complex methods and provided valuable guidance for future research.

Table 5 presents descriptive statistics for DMT and DTR, all units being degrees Celsius. The sample size for both DMT and DTR is 123,987 observations. The mean value for DMT is 15.01°Cwith a standard deviation of 10.94°C, reaching a minimum value of −15°C and a maximum value of 32.5°C. As for the DTR, its mean value is 9.22°C, with a standard deviation of 3.82°C, a minimum value of 0°C, and a maximum value reaching 32°C.

**Table 5:** Descriptive statistics of independent variables.

Variable	Obs	Mean	Std. dev.	Min	Max
DMT	123,987	15.01208	10.93858	−15	32.5
DTR	123,987	9.219015	3.821875	0	32

### 3.3 Analytical method

For the baseline panel regression, three indices of negative mood are used as dependent variables, while the DMT and DTR serve as independent variables. Both fixed effects and random effects models are constructed for analysis. The formula for calculating the fixed effects model is generally represented as:


$$\left\{ \begin{array}{l} \text{Depressio}n_{\text{it}}=\alpha_{i}+\beta_{1}{\text{DMT}}_{\text{it}}+\beta_{2}{\text{DTR}}_{\text{it}}+\epsilon_{\text{it}}\\ \text{Anxiet}y_{\text{it}}=\alpha_{i}+\beta_{3}{\text{DMT}}_{\text{it}}+\beta_{4}{\text{DTR}}_{\text{it}}+\epsilon_{\text{it}}\\ \text{Lonelines}s_{\text{it}}=\alpha_{i}+\beta_{5}{\text{DMT}}_{\text{it}}+\beta_{6}{\text{DTR}}_{\text{it}}+\epsilon_{\text{it}} \end{array}\right.$$

Where $\text{Depressio}n_{\text{it}}$ represents the depression level of residents in city i at time t, $\text{Anxiet}y_{\text{it}}$ represents the anxiety level of residents in city i at time t, $\text{Lonelines}s_{\text{it}}$ represents the loneliness level of residents in city i at time t; $\alpha_{i}$ is the unique fixed effect for each city, capturing those time-invariant specific characteristics of the city; $\beta_{1}$ to $\beta_{6}$ are, respectively, the influence coefficients of DMT and DTR on negative emotions; $\epsilon_{\text{it}}$ is the error term, representing the part not explained by the model. The calculation formula for random effects is:


$$\left\{ \begin{array}{l} \text{Depressio}n_{\text{it}}=\beta_{0}+\beta_{1}{\text{DMT}}_{\text{it}}+\beta_{2}{\text{DTR}}_{\text{it}}+u_{i}+\epsilon_{\text{it}}\\ \text{Anxiet}y_{\text{it}}=\beta_{0}+\beta_{3}{\text{DMT}}_{\text{it}}+\beta_{4}{\text{DTR}}_{\text{it}}+u_{i}+\epsilon_{\text{it}}\\ \text{Lonelines}s_{\text{it}}=\beta_{0}+\beta_{5}{\text{DMT}}_{\text{it}}+\beta_{6}{\text{DTR}}_{\text{it}}+u_{i}+\epsilon_{\text{it}} \end{array}\right.$$

Where $u_{i}$ is an individual-specific random effect, assuming it is uncorrelated with the independent variables and independently identically distributed among individuals, usually assumed $u_{i}∼N(0,\sigma_{\epsilon }^{2})$; $\beta_{0}$ is the intercept; $\epsilon_{\text{it}}$ is the error term, representing the part not explained by the model, assuming $\epsilon ∼N(0,\sigma_{\epsilon }^{2})$, and it is uncorrelated with $u_{i}$.

Next, Hausman test method is used to compare the estimation results between fixed effects models and random effects models, to determine which model is more suitable for the data. The null hypothesis (H0) of this test assumes that there is no systematic difference between the random effects estimator and the fixed effects estimator, i.e., the random effects model is valid. The test statistic (H) can be expressed as:


$$H=({\hat{\beta }}_{\text{FE}}-{\hat{\beta }}_{\text{RE}})\prime {\left[\text{Var}({\hat{\beta }}_{\text{FE}})-\text{Var}({\hat{\beta }}_{\text{RE}})\right]}^{-1}({\hat{\beta }}_{\text{FE}}-{\hat{\beta }}_{\text{RE}})$$

Where ${\hat{\beta }}_{\text{FE}}$ is the vector of estimated coefficients for the fixed effects model, ${\hat{\beta }}_{\text{FE}}$ is the vector of estimated coefficients for the random effects model, $\text{Var}({\hat{\beta }}_{\text{FE}})$ and $\text{Var}({\hat{\beta }}_{\text{RE}})$ are the variance–covariance matrices of the estimated coefficients for the fixed effects and random effects models, respectively. Since the calculated H value in this study is greater than the critical value at the given significance level, the null hypothesis is rejected, and the fixed-effect model is selected as the baseline model.

To verify the robustness of the baseline model, Huber-White heteroscedasticity-consistent standard errors, also known as robust standard errors, are used for estimation. For the linear regression model $y_{i}=x_{i}^{\prime }\beta +\epsilon_{i}$, where i=1,2,…,n represents the observations, $x_{i}^{\prime }$ is the explanatory variable vector, β is the parameter vector, and $\epsilon_{i}$ is the error term. In the presence of heteroscedasticity, the robust variance–covariance matrix can be calculated in the following way:


$${\hat{V}}_{\text{robust}}={\left(X\prime X\right)}^{-1}(\sum_{i=1}^{n}{\hat{u}}_{i}^{2}x_{i}{xi}_{\prime }){\left(X\prime X\right)}^{-1}$$

Where X is the design matrix, which includes all explanatory variables of the observations; ${\hat{u}}_{i}^{2}=y_{i}-{x\prime }_{i}\hat{\beta }$ is the residual of the i-th observation; $\hat{\beta }$ is the OLS estimate of the regression coefficient; ${\left(X\prime X\right)}^{-1}$ is part of the variance–covariance matrix under ordinary least squares (OLS). This formula provides a correction for the standard errors of the original OLS estimates, making them valid even in the presence of heteroscedasticity. In particular, this method does not rely on the assumption of homoscedasticity and can provide more accurate standard errors for parameter estimates.

Due to the fact that the DTR as one of the independent variables is not significant in the coefficient under robust standard error tests, threshold regression is further adopted to analyze non-linear impacts. With DTR as the threshold variable and DMT as the explanatory variable, the regression equation for solving loneliness is:


$${\text{loneliness}}_{i}=\{\begin{array}{ll} \beta_{0}+\beta_{1}{\text{DMT}}_{i}+\epsilon_{i} & \text{if}\;{\text{DTR}}_{i}\leq \gamma \\ \alpha_{0}+\alpha_{1}{\text{DMT}}_{i}+\epsilon_{i} & \text{if}\;{\text{DTR}}_{i}>\gamma \end{array}$$

Where ${\text{loneliness}}_{i}$ represents the loneliness of residents in city i; ${\text{DMT}}_{i}$ represents DMT corresponding to city i; ${\text{DTR}}_{i}$ is the DTR corresponding to city i; γ is the threshold parameter, indicating the critical value of DTR, beyond which the impact of DTR on loneliness changes; $\beta_{0},\beta_{1}$ and $\alpha_{0},\alpha_{1}$ are two sets of different regression coefficients, corresponding to the cases where DTR is less than or equal to and greater than the threshold γ, respectively; $\epsilon_{i}$ is the error term.

Before conducting further heterogeneity analysis, it is necessary to determine the criteria for dividing seasonal periods. In this study, to smooth out the fluctuations in DMT data and identify long-term trends, we calculated a 5-day moving average. Specifically, for each day’s DMT data, its moving average value (moving_avg) is calculated using the following formula:


$${\text{MovingAvg}}_{t}=\frac{1}{5}\sum_{i=t-2}^{t+2}{\text{DMT}}_{i}$$

Where ${\text{MovingAvg}}_{t}$ represents the moving average temperature of each day, obtained by averaging the DMT values of that day and the 2 days before and after it. Considering that at the beginning and end of the data series, a complete 5-day window may not be available, a center-symmetric rolling window (center = True) was used, and windows with fewer than 5 values were applied when necessary to fill in NaN values, ensuring that even edge data are processed. Further, to fill any remaining NaN values in the ‘moving_avg’ column, another calculation of the rolling average was performed, this time allowing a minimum window size of 1 (min_periods = 1), to ensure all points could be filled.

Subsequently, based on the moving average temperature, we conducted seasonal categorization. The specific classification criteria are as follows:


$$\text{Season}=\{\begin{array}{ll} \text{Hottest}, & {\text{if MovingAvgFilled}}_{t}\geq 22\\ \text{Coldest}, & {\text{if MovingAvgFilled}}_{t}\leq 10\\ \text{Transitional}, & \text{otherwise} \end{array}$$

Where ${\text{MovingAvgFilled}}_{t}$ refers to the moving average temperature values filled in after the aforementioned steps. According to the QX/T 152–2012 standard, daily temperature environments are categorized into three types: When the moving average temperature reaches or exceeds 22 degrees Celsius, it is defined as category ‘Hottest’, representing high-temperature seasons; when the moving average temperature is less than or equal to 10 degrees Celsius, it is defined as category ‘Coldest’, indicating low-temperature seasons; all other cases are classified as ‘Transitional’, i.e., transitional seasons. This classification method facilitates a better understanding and analysis of trends in emotional variables under different temperature conditions.

After reasonable grouping, Analysis of Variance (ANOVA) can be used to compare differences between groups. ANOVA is utilized to compare the means of two or more groups to determine if there are any statistically significant differences among them. Its core calculation is based on the Total Sum of Squares (SST), Between-group Sum of Squares (SSB), and Within-group Sum of Squares (SSW). The formulas for these calculations are as follows:


$$F=\frac{\frac{\sum_{j=1}^{k}n_{j}{\left({\bar{y}}_{j}-\bar{y}\right)}^{2}}{k-1}}{\frac{\sum_{j=1}^{k}\sum_{i=1}^{n_{j}}{\left(y_{\text{ij}}-{\bar{y}}_{j}\right)}^{2}}{N-k}}$$

Where F is the statistic used to test whether the differences between groups are significantly greater than the differences within groups. The numerator represents the mean square between groups, where k is the number of groups, $n_{j}$ is the sample size of the j-th group, ${\bar{y}}_{j}$ is the average value of the j-th group, and $\bar{y}$ is the overall average value of all observations. The denominator is the mean square within groups, where $y_{\text{ij}}$ is the j-th observation in the j-th group, and N is the total number of observations.

Subsequently, Tukey’s HSD test method is used to determine whether there are significant differences between any two groups, with the calculation formula as follows:


$$\text{HSD}=q_{\alpha }(k,N-k)\cdot \sqrt{\frac{\frac{\sum_{j=1}^{k}\sum_{i=1}^{n_{j}}{\left(y_{\text{ij}}-{\bar{y}}_{j}\right)}^{2}}{N-k}}{n}}n$$

Where $\text{HSD}=q_{\alpha }(k,N-k)$ is the critical value obtained from the studentized range distribution table based on the significance level *α*, the number of groups k, and the degrees of freedom N−k. $\text{MSW}=\frac{\sum_{j=1}^{k}\sum_{i=1}^{n_{j}}{\left(y_{\text{ij}}-{\bar{y}}_{j}\right)}^{2}}{N-k}$ is the mean square within groups, where nn is the sample size of each group.

Finally, within the divided 5×3=15 groups, the benchmark panel regression estimation method is reused to compare the differences in the degree of influence of independent variables on dependent variables between groups.

## 4 Results

### 4.1 Baseline model

The estimation results of fixed effects and random effects are shown in Table 6. The values within parentheses are t-statistics, with * indicating significance at the 0.1 level, ** at the 0.05 level, and *** at the 0.01 level. This notation applies to all subsequent tables and will not be repeated. The benchmark regression results indicate that the independent variables, DMT and DTR, have significant impacts on three types of negative emotions. Overall, there is a significant positive correlation between DMT and negative emotions, meaning higher temperatures are more likely to trigger negative emotions. Conversely, there is a significant negative correlation between DTR and negative emotions, implying that greater fluctuations in temperature are associated with a lower probability of experiencing negative emotions.

**Table 6:** Benchmark panel regression.

Variable	Fixed effect (better)			Random effect		
	Depression	Anxiety	Loneliness	Depression	Anxiety	Loneliness
DMT	0.2271***	0.1876***	0.1959***	0.2274***	0.1879***	0.1965***
	(21.1822)	(16.7083)	(11.5825)	(21.2063)	(16.7374)	(11.6192)
DTR	−0.3035***	−0.3119***	−0.1541**	−0.3045***	−0.3132***	−0.1563**
	(−9.3775)	(−9.2019)	(−3.0184)	(−9.4081)	(−9.2400)	(−3.0603)
_cons	107.8454***	107.6664***	148.5923***	107.8457***	107.6778***	148.5825***
	(320.5324)	(305.5729)	(279.9099)	(15.9397)	(16.9057)	(19.5698)
N	123,987	123,987	123,987	123,987	123,987	123,987
r2_w	0.0040	0.0027	0.0011	0.0040	0.0027	0.0011
r2_b	0.2369	0.2705	0.2426	0.2370	0.2705	0.2427
r2_o	0.0631	0.0671	0.0373	0.0632	0.0671	0.0374

To determine whether to use a fixed effects model or a random effects model, a Hausman test was conducted. The test results are shown in Table 7. The Chi-Squared Statistic is 9.46 with 2 Degrees of Freedom, and the *p*-value is 0.0088. Since the *p*-value is less than the commonly used significance level (e.g., 0.05), we reject the null hypothesis, concluding that there are systematic differences between the coefficients. Although the differences in coefficients are numerically small, the statistical significance indicates that there is a correlation between individual effects and the explanatory variables, necessitating the use of a fixed effects model to address this endogeneity issue.

**Table 7:** Hausman test results.

Variable	Fixed effect(b)	Random effect(B)	Difference(b-B)	St. error
DMT	0.1959312	0.1965401	−0.0006089	(0.0001815)
DTR	−0.1541387	−0.1562625	0.0021237	(0.0006867)

### 4.2 Robust model

When the fixed effects model is re-estimated using the robust standard errors approach (see Table 8), the results remain largely consistent with those of the baseline model. The exception is that the significance of the coefficient for the effect of daily temperature differences on feelings of loneliness disappears. All other independent variables maintain their significant and reliable impact on the dependent variable.

**Table 8:** Robust standard errors fixed effects estimation.

Variable	Depression	Anxiety	Loneliness
DMT	0.2271***	0.1876***	0.1959***
	(6.9429)	(5.8613)	(4.1453)
DTR	−0.3035**	−0.3119**	−0.1541
	(−3.5321)	(−3.1366)	(−1.1297)
_cons	107.8454***	107.6664***	148.5923***
	(123.1279)	(110.3920)	(135.7255)
N	123,987	123,987	123,987
r2_w	0.0040	0.0027	0.0011
r2_b	0.2369	0.2705	0.2426
r2_o	0.0631	0.0671	0.0373

### 4.3 Threshold analysis

Given that the impact of DTR on loneliness did not pass the robust standard error test during robustness evaluation (see Table 8), this study further explores the nonlinear effects of DTR on individual loneliness using a panel threshold regression model, while also considering the role of DMT as a control variable. Table 9 presents the main findings of the model. The study reveals that when DTR exceeds an estimated threshold of 14.0 (with a confidence interval of [12.0, 15.0]), there is a significant change in the mechanism through which DTR affects loneliness. Specifically, when DTR is less than or equal to 14.0, there is a positive and statistically significant relationship between DMT and loneliness (coefficient = 0.376451, *t*-value = 6.98, *p* < 0.001). However, when DTR exceeds 14.0, this relationship becomes insignificant (coefficient = 0.0034506, *t*-value = 0.03, *p* = 0.975), suggesting that larger DTR may weaken the impact of DMT on loneliness. Additionally, although the overall explanatory power of the model is limited (Within R-squared = 0.0028), the explanatory power for variations between cities is relatively high (Between R-squared = 0.5803), indicating that differences between cities might be a significant factor influencing loneliness.

**Table 9:** Threshold analysis.

Parameter	Coefficient	Standard error	*t*-value	*p*-value	95% confidence interval
Threshold	14.0000	–	–	–	[12.0000, 15.0000]
DMT (DTR ≤ 14)	0.376451	0.053924	6.98	<0.001	[0.270756, 0.4821464]
DMT (DTR > 14)	0.0034506	0.1082925	0.03	0.975	[−0.2088117, 0.2157129]

### 4.4 Regional heterogeneity regression

Table 10 shows the regression results of a cross-group analysis involving three emotions (depression/anxiety/loneliness) × five regions (extremely cold/cold/cold summer hot winter/hot summer warm winter/mild) × two estimation methods (baseline/robust). Overall, the DMT has a more robust impact on negative emotions compared to DTR. Regarding the direction of the impact of independent variables on dependent variables, the findings for depression and anxiety across all climate regions (see Table 10) are consistent with those from the full-region baseline model (see Table 6). However, in extremely cold regions, the effect of DTR on loneliness reverses; that is, greater temperature fluctuations lead to an increased likelihood of experiencing loneliness. In robust estimations, the impact of DMT on the dependent variable tends to be insignificant more easily in the coldest region (extremely cold) compared to the other four relatively warmer regions.

**Table 10:** Regional heterogeneity analysis of negative emotions.

Method		Region	Sever cold	Cold	Hot Sum. Cold Win.	Hot Sum. Warm Win.	Temperate
Baseline regression	Depression	DMT	0.1222***	0.1765***	0.2688***	0.6026***	0.3210***
			(5.3979)	(11.2830)	(11.5350)	(13.9963)	(7.6208)
		DTR	−0.2536**	−0.2093***	−0.1779**	−0.4759***	−0.9652***
			(−2.5097)	(−4.2984)	(−2.6677)	(−4.7018)	(−10.5184)
	Anxiety	DMT	0.0641**	0.1251***	0.2685***	0.5794***	0.2828***
			(2.5825)	(7.6885)	(10.7234)	(13.1458)	(6.6914)
		DTR	−0.2210**	−0.1354**	−0.2586***	−0.7056***	−0.9362***
			(−1.9933)	(−2.6731)	(−3.6088)	(−6.8092)	(−10.1706)
	Loneliness	DMT	0.0025	0.1783***	0.2655***	0.6162***	0.2433***
			(0.0834)	(7.5250)	(6.6732)	(8.4473)	(4.0200)
		DTR	0.7300***	0.0220	−0.1442	−1.2158***	−0.6409***
			(5.4633)	(0.2983)	(−1.2664)	(−7.0897)	(−4.8618)
VCE Robust	Depression	DMT	0.1222*	0.1765**	0.2688**	0.6026**	0.3210
			(4.2899)	(3.7882)	(4.9156)	(4.5601)	(1.9157)
		DTR	−0.2536	−0.2093	−0.1779	−0.4759	−0.9652**
			(−0.7460)	(−1.7595)	(−1.7460)	(−1.5465)	(−3.3277)
	Anxiety	DMT	0.0641	0.1251**	0.2685***	0.5794**	0.2828*
			(1.4496)	(3.0417)	(5.7357)	(4.9288)	(2.9881)
		DTR	−0.2210	−0.1354	−0.2586	−0.7056**	−0.9362**
			(−0.4913)	(−0.8005)	(−1.8349)	(−4.0276)	(−4.2209)
	Loneliness	DMT	0.0025	0.1783**	0.2655**	0.6162*	0.2433**
			(0.0539)	(2.5268)	(3.2874)	(2.2700)	(4.7391)
		DTR	0.7300	0.0220	−0.1442	−1.2158	−0.6409
			(1.7075)	(0.1525)	(−0.7053)	(−2.0705)	(−1.7698)

### 4.5 Regional-seasonal variance analysis

Independent single-factor ANOVAs were conducted across five climate regions to explore the impact of seasonal changes on three types of negative emotions. The results showed significant seasonal effects in all regions (all *p* < 0.05). Table 11 presents the detailed outcomes of the ANOVA analyses for each region.

**Table 11:** ANOVA results.

Mood	Region	df (model)	df (residual)	F	*p*	Sig
Depression	Sever Cold	2	11,916	21.70	0.0000	YES
	Cold	2	44,162	57.49	0.0000	YES
	Hot Sum. Cold Win.	2	31,873	135.13	0.0000	YES
	Hot Sum. Warm Win.	2	20,032	65.29	0.0000	YES
	Temperate	2	15,989	5.28	0.0051	YES
Anxiety	Sever Cold	2	11,916	10.04	0.0000	YES
	Cold	2	44,162	74.72	0.0000	YES
	Hot Sum. Cold Win.	2	31,873	130.19	0.0000	YES
	Hot Sum. Warm Win.	2	20,032	55.91	0.0000	YES
	Temperate	2	15,989	9.62	0.0001	YES
Loneliness	Sever Cold	2	11,916	7.06	0.0009	YES
	Cold	2	44,162	40.45	0.0000	YES
	Hot Sum. Cold Win.	2	31,873	109.75	0.0000	YES
	Hot Sum. Warm Win.	2	20,032	84.52	0.0000	YES
	Temperate	2	15,989	11.30	0.0000	YES

Tukey’s HSD pairwise comparisons were further conducted, and the results are shown in Table 12. The “region-season” heterogeneity analysis revealed the following trends:

- For depressive mood, significant inter-group differences were observed during: cold season in severely cold regions, all seasons in cold regions, all seasons in regions with hot summers and cold winters, hot season in regions with hot summers and warm winters, and hot season in temperate regions.
- The heterogeneity pattern for anxiety was almost identical to that of depressive mood.
- For feelings of loneliness, significant inter-group differences were found during: cold season in severely cold regions, transitional seasons in cold regions, all seasons in regions with hot summers and cold winters, all seasons in regions with hot summers and warm winters, and cold season in temperate regions.

**Table 12:** Tukey’s HSD pairwise comparisons.

Mood	Region	Groups	Contrast	Std. err.	t	P > |t|	95% cnf. interval		Difference
Depression	Sever Cold	Cold vs. Hot	−5.280998	0.9287488	−5.69	0.000	−7.45798	−3.104016	Cold
		Tran vs. Hot	−1.463429	0.8984413	−1.63	0.233	−3.56937	0.6425115	
		Tran vs. Cold	3.817569	0.7050445	5.41	0.000	2.164949	5.470189	
	Cold	Cold vs. Hot	−3.000999	0.7410795	−4.05	0.000	−4.737868	−1.264131	All
		Tran vs. Hot	3.661276	0.7004568	5.23	0.000	2.019615	5.302937	
		Tran vs. Cold	6.662275	0.6257668	10.65	0.000	5.195665	8.128885	
	Hot Summer Cold Winter	Cold vs. Hot	−7.440288	0.6965753	−10.68	0.000	−9.072851	−5.807724	All
		Tran vs. Hot	2.489071	0.6397897	3.89	0.000	0.9895955	3.988547	
		Tran vs. Cold	9.929359	0.6109795	16.25	0.000	8.497406	11.36131	
	Hot Summer Warm Winter	Cold vs. Hot	8.448866	0.9679526	8.73	0.000	6.180106	10.71763	Hot
		Tran vs. Hot	9.44299	0.8685556	10.87	0.000	7.407204	11.47878	
		Tran vs. Cold	0.9941234	0.8963687	1.11	0.508	−1.106853	3.0951	
	Temperate	Cold vs. Hot	4.523267	1.528704	2.96	0.009	0.9401065	8.106427	Hot
		Tran vs. Hot	3.702438	1.27227	2.91	0.010	0.7203407	6.684536	
		Tran vs. Cold	−0.8208285	1.223605	−0.67	0.780	−3.68886	2.047203	
Anxiety	Sever Cold	Cold vs. Hot	−4.088108	1.027937	−3.98	0.000	−6.497587	−1.678629	Cold
		Tran vs. Hot	−1.31247	0.9943929	−1.32	0.381	−3.648098	1.013604	
		Tran vs. Cold	2.77086	0.703417	3.55	0.001	0.914739	4.599977	
	Cold	Cold vs. Hot	−1.869996	0.7074438	−2.64	0.022	−3.528033	−0.2119597	All
		Tran vs. Hot	5.129975	0.6686649	7.67	0.000	3.562825	6.697126	
		Tran vs. Cold	6.999971	0.5973649	11.72	0.000	5.599927	8.400016	
	Hot Summer Cold Winter	Cold vs. Hot	−5.862844	0.7365897	−7.96	0.000	−7.58919	−4.136499	All
		Tran vs. Hot	4.554228	0.6765421	6.73	0.000	2.968616	6.13984	
		Tran vs. Cold	10.41707	0.6460769	16.12	0.000	8.902862	11.93128	
	Hot Summer Warm Winter	Cold vs. Hot	7.944835	0.9654678	8.23	0.000	5.681899	10.20777	Hot
		Tran vs. Hot	8.65069	0.866326	9.99	0.000	6.62013	10.68125	
		Tran vs. Cold	0.705855	0.8940677	0.79	0.710	−1.389728	2.801438	
	Temperate	Cold vs. Hot	4.465024	1.508797	2.96	0.009	0.9285257	8.001523	Hot
		Tran vs. Hot	5.499778	1.255702	4.38	0.000	2.556514	8.443041	
		Tran vs. Cold	1.034753	1.207671	0.86	0.668	−1.795929	3.865436	
Loneliness	Sever Cold	Cold vs. Hot	−4.384877	1.262744	−3.47	0.002	−7.344741	−1.425013	Cold
		Tran vs. Hot	−1.732893	1.221537	−1.42	0.331	−4.596168	1.130383	
		Tran vs. Cold	2.651984	0.9585912	2.77	0.016	0.4050524	4.898917	
	Cold	Cold vs. Hot	0.32658	0.9201585	0.35	0.933	−1.829996	2.483156	Tran
		Tran vs. Hot	6.298261	0.8697196	7.24	0.000	4.259899	8.336624	
		Tran vs. Cold	5.971681	0.776981	7.69	0.000	4.150671	7.792692	
	Hot Summer Cold Winter	Cold vs. Hot	6.364561	1.06736	5.96	0.000	3.862988	8.866135	All
		Tran vs. Hot	14.20391	0.9803481	14.49	0.000	11.90627	16.50155	
		Tran vs. Cold	7.839349	0.9362023	8.37	0.000	5.645171	10.03353	
	Hot Summer Warm Winter	Cold vs. Hot	18.16197	1.530972	11.86	0.000	14.57357	21.75038	All
		Tran vs. Hot	14.81317	1.37376	10.78	0.000	11.59325	18.0331	
		Tran vs. Cold	−3.348801	1.417751	−2.36	0.048	−6.671832	−0.0257694	
	Temperate	Cold vs. Hot	6.305099	1.730378	3.64	0.001	2.249231	10.36097	Cold
		Tran vs. Hot	−0.0903579	1.440113	−0.06	0.998	−3.465868	3.285152	
		Tran vs. Cold	−6.395457	1.385029	−4.62	0.000	−9.641853	−3.149061	

### 4.6 Regional-seasonal subgroup regression

Finally, a fixed-effects panel regression similar to the baseline model was employed to analyze the conditions of 45 subgroups. Table 13 provides a detailed overview of the seasonal heterogeneity characteristics across different climate zones for examination. The heterogeneity is notably complex, with varying impacts of different climate zones and seasons on emotional states presenting several key patterns:

- Pattern One involves the bidirectional regulatory effect of temperature changes on mood. For instance, in severely cold regions during the cold season, an increase in DMT is significantly positively correlated with depression probability (coefficient 0.2877***), suggesting that low-temperature environments may reduce depression risk through mechanisms such as increased social interaction needs during cold seasons, whereas higher temperatures may exacerbate depression. However, an increase in DTR is significantly negatively correlated with depression probability (coefficient −0.8419***), indicating that larger DTR might mitigate depression by regulating diurnal activities or fluctuating temperatures. Similarly, in severely cold regions during transitional seasons, an increase in DTR is significantly positively correlated with loneliness probability (coefficient 1.1157***), suggesting that greater temperature variability could intensify feelings of loneliness, while an increase in DMT is negatively correlated with loneliness probability (−0.2703***), implying that lower temperatures might reduce loneliness.
- Pattern Two highlights the widespread suppressive effect of temperature variation on anxiety. The negative correlation between DTR and anxiety probability is significant across most regions. For example, in severely cold regions during the cold season, an increase in DTR is significantly negatively correlated with anxiety probability (coefficient −0.9143***), indicating that larger DTR might alleviate anxiety by promoting circadian rhythm balance or reducing long-term exposure to low temperatures. A similar pattern is observed in temperate regions during the cold season (DTR coefficient −1.5071***), but an increase in DMT is positively correlated with anxiety probability (coefficient 1.1690***), suggesting that while low temperatures exacerbate anxiety in temperate regions, larger DTR can mitigate this impact.
- Pattern Three involves the interaction effects between seasons and regions. For instance, in temperate regions during the cold season, an increase in DMT is significantly positively correlated with depression probability (coefficient 0.6774***), but an increase in DTR is significantly negatively correlated with depression probability (coefficient −1.2144***), indicating that the direction of the negative emotional impact of the same meteorological parameter can be entirely opposite across different regions. Additionally, during transitional seasons, an increase in DTR in severely cold regions is positively correlated with loneliness probability (coefficient 1.1157***), while it is significantly negatively correlated with loneliness in warm summer and mild winter regions (coefficient −1.4641***), highlighting the differential psychological impacts of climatic fluctuations across various regions during transitional seasons.
- Pattern Four illustrates the reversal of effects between hot and cold seasons. For example, in severely cold regions, an increase in DMT during the hot season is negatively correlated with anxiety probability (coefficient −0.4574**), while in the cold season, it is positively correlated with anxiety probability (0.4147***). This suggests that the impact of climate on mental health not only depends on absolute temperature levels but also on whether these temperature changes align with people’s expectations and adaptation patterns in different regions.
- Pattern Five emphasizes the complexity of transitional seasons. The impact of meteorological parameters on negative emotions during transitional seasons tends to be more pronounced. For example, in warm summer and mild winter regions, an increase in DTR is significantly negatively correlated with loneliness probability (coefficient −1.4641***), indicating that larger DTR during transitional seasons might decrease loneliness by promoting adaptive behaviors (such as adjusting routines). Conversely, in severely cold regions during transitional seasons, an increase in DTR is positively correlated with loneliness probability (coefficient 1.1157***), possibly due to reduced outdoor social opportunities in low-temperature environments, where larger temperature variations could exacerbate loneliness.

**Table 13:** Regional-seasonal subgroup regression.

Season	Region	Hot	Cold	Tran	Hot	Cold	Tran	Hot	Cold	Tran
		Depression			Anxiety			Loneliness		
Sever Cold	DMT	−0.0464	0.2877***	−0.0649	−0.4574**	0.4147***	−0.2160***	0.1774	−0.0940	−0.2703***
		(−0.2705)	(5.2368)	(−1.4133)	(−2.4818)	(6.8307)	(−4.3022)	(0.7717)	(−1.3006)	(−4.4654)
	DTR	0.0203	−0.8419***	−0.0023	−0.0341	−0.9143***	0.0922	0.4158	0.1381	1.1157***
		(0.0759)	(−4.6383)	(−0.0161)	(−0.1187)	(−4.5590)	(0.5916)	(1.1600)	(0.5785)	(5.9375)
Cold	DMT	−0.1076	0.2241***	0.0104	−0.0548	0.2834***	−0.0366	0.3189**	0.4259***	0.1457**
		(−1.1651)	(4.6711)	(0.3177)	(−0.6018)	(5.5209)	(−1.0702)	(2.4914)	(5.8893)	(2.8147)
	DTR	0.0622	−0.3989***	−0.2338***	0.1491	−0.5560***	−0.0855	−0.1205	−0.4112**	0.1757
		(0.5608)	(−4.2858)	(−3.3864)	(1.3631)	(−5.5837)	(−1.1843)	(−0.7839)	(−2.9320)	(1.6073)
Hot Summer Cold Winter	DMT	−0.0451	0.2538***	0.1148**	0.2432**	0.2048**	0.1393**	0.2786*	0.8292***	0.1845**
		(−0.4991)	(4.1586)	(2.6686)	(2.6353)	(3.1283)	(2.9391)	(1.8993)	(7.8619)	(2.4679)
	DTR	0.0992	−0.1205	−0.3870***	0.2113	−0.1681	−0.5972***	−0.9566***	0.2539	−0.4433**
		(0.6303)	(−1.0120)	(−4.0412)	(1.3155)	(−1.3165)	(−5.6607)	(−3.7475)	(1.2342)	(−2.6648)
Hot Summer Warm Winter	DMT	0.2027	1.2576***	0.0408	−0.0068	1.5363***	−0.1996*	−0.5771	1.0434***	0.4529**
		(0.7993)	(8.8290)	(0.3752)	(−0.0271)	(10.0645)	(−1.8075)	(−1.4705)	(4.1875)	(2.3818)
	DTR	−0.5436**	−0.4748**	−0.1586	−0.4201	−1.0125***	−0.2594*	−0.5295	−0.5768*	−1.4641***
		(−2.0200)	(−2.7087)	(−1.0307)	(−1.5846)	(−5.3909)	(−1.6609)	(−1.2719)	(−1.8812)	(−5.4437)
Temperate	DMT	−0.3067	0.6774***	0.3172***	−0.3510	1.1690***	0.0995	−1.1006**	0.7585**	0.1747
		(−1.0877)	(3.9995)	(4.1888)	(−1.2881)	(6.5662)	(1.3201)	(−2.8149)	(2.9734)	(1.6245)
	DTR	−0.1555	−1.2144***	−1.1512***	−0.0613	−1.5071***	−1.0457***	−1.7885***	−0.4085	−0.5124**
		(−0.5276)	(−6.1675)	(−9.6926)	(−0.2154)	(−7.2816)	(−8.8488)	(−4.3768)	(−1.3775)	(−3.0376)

**Figure 5:**
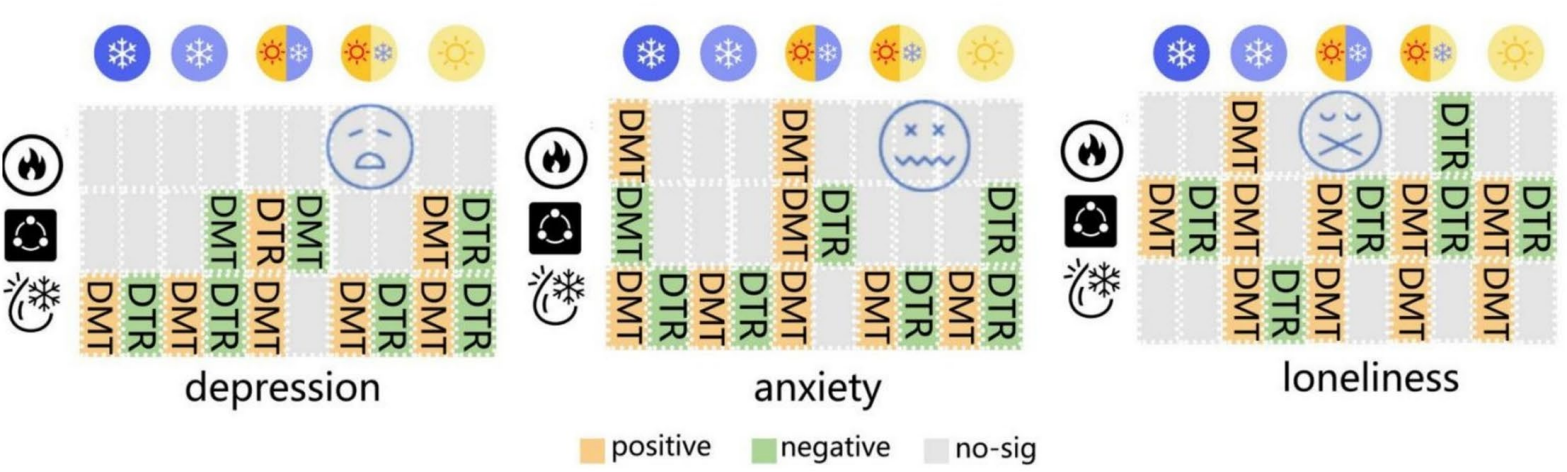
Regional-seasonal heterogeneity.

Figure 5 summarizes the test results, with the horizontal axis representing climatic regions and the vertical axis representing seasonal periods. The left side shows the daily average temperature, while the right side shows the daily temperature difference. Orange signifies significant positive correlation (*p* < 0.05), green indicates significant negative correlation (*p* < 0.05), and gray denotes non-significant correlation (*p* ≥ 0.05).

## 5 Discussion and conclusion

This study delves into the impact of temperature variations on public mood, with a particular focus on an exhaustive analysis of data spanning 11 years across Chinese provincial capitals. In light of our data analysis results, we aim to explore the following three questions.

One of the primary valuable conclusions of this research is the validation of the relationship between temperature and negative emotions. The findings indicate a significant positive correlation between DMT and negative emotions, suggesting that higher temperatures are more likely to trigger negative moods. This discovery supports earlier studies on how high temperatures exacerbate negative feelings, such as the works of Bell & Baron and Howarth & Hoffman.

Regarding the mechanisms by which temperature affects mood, several physiological studies provide insightful explanations. Lowry et al. (49) discussed the role of brain serotonergic neurons in emotion regulation and proposed that temperature changes might alter mood states by influencing serotonin levels, providing a neurochemical basis for understanding the relationship between temperature and mood. Osimo et al. (50) meta-analysis further indicated that inflammation triggered by temperature variations might be associated with the onset and progression of depression, offering a biological perspective on how weather impacts mood and highlighting the profound potential effects of environmental temperature changes on mental health. Nakata et al. (51) utilized event-related potentials (ERP) to study the impact of passive heat stress and recovery on human cognitive function, demonstrating how temperature changes specifically alter brain activity patterns, thereby affecting cognitive processing. These studies collectively suggest that temperature changes can significantly impact human emotions and cognition through various mechanisms, including neurochemical pathways, inflammatory responses, and direct alterations in brain activity patterns.

Additionally, I propose a bold hypothesis to explain why our findings diverge from those of Stephens-Davidowitz: this discrepancy might be attributed to cultural differences, particularly those inherent in East Asian cultures. A study by Hurley et al. (52) in Singapore demonstrated that cultural backgrounds significantly influence the way emotions are expressed, with factors like collectivism values and face-saving considerations affecting people’s openness when expressing emotions. In China, to maintain social harmony, individuals may be more inclined to suppress their anger and dissatisfaction, which could account for the divergence in findings between studies conducted in China and the United States. With the continuous exacerbation of the El Niño phenomenon (53), numerous studies have established a link between global warming and increased mortality rates (54–56). The author speculates that there may be a threshold for human tolerance to temperature, beyond which extreme weather conditions not only fail to promote well-being but also exacerbate negative emotions. Liu (57) points out that while warm climates encourage more outdoor social activities, they can also increase social pressure, leading to an accumulation of negative emotions. Zhu et al. (58) further elaborate that the impact of emotional suppression on health varies significantly across different cultural contexts. For East Asians, emotional suppression does not correlate with poor sleep quality as it does in Western cultures, explaining why Chinese people exhibit different responses to negative emotions compared to Americans. Chan et al. (59) conducted cluster analysis on emotional subtypes among patients with depression, revealing that long-term suppression of inner emotions, such as anger, could lead to the development of depressive and other negative emotional states, emphasizing the importance of emotional expression. These viewpoints remain unverified hypotheses and inferences drawn from literature, requiring more detailed mechanism studies and comparative experiments for validation.

Secondly, this study found that daily temperature range (DTR) is significantly negatively correlated with negative emotions, indicating that the greater the temperature fluctuation, the less likely individuals are to experience negative emotions. One possible explanation for this phenomenon is that larger temperature variations prompt people to pay more attention to weather changes and take corresponding preventive measures, thereby alleviating psychological stress caused by extreme weather. However, this conclusion contrasts with some earlier studies. For instance, Sanders and Brizzolara and Huibers et al. did not find a significant relationship between weather conditions and emotions. Moreover, although Denissen et al. indicated that temperature, wind force, and sunlight have significant effects on negative emotions, they did not delve into the role of daily temperature ranges. Therefore, this study provides a new perspective on understanding how temperature fluctuations influence human emotions.

Additionally, research on regional-seasonal variability has revealed significant differences in the impact of temperatures on negative emotions across different climatic regions. In colder regions (extremely cold/cold), the effect of daily temperature range on feelings of loneliness is reversed, meaning that the greater the temperature fluctuation, the more likely it is to trigger feelings of loneliness. This finding differs from previous research on the impact of temperature changes on emotions. For example, Keller et al. emphasized the spring warmth effect, and Lee et al. focused on productivity increases under high-temperature conditions without considering the particularities of cold regions. This study reveals that within specific climatic regions, seasonal factors such as the cold season in extremely cold areas or the hot season in temperate regions show more pronounced negative emotional fluctuations, suggesting the need for differentiated mental health intervention strategies tailored to different climatic regions and seasons.

In summary, this study employs econometric methods such as panel regression analysis, robustness checks, and heterogeneity comparisons, in conjunction with Baidu Index data serving as an emotional indicator, to explore the macro mechanisms by which temperature variations influence public emotions. It offers new insights into how temperature affects negative emotions among residents in provincial capital cities of China. Key findings include:

- A significant positive correlation between daily average temperature and negative emotions;
- A negative correlation between daily temperature range (DTR) and negative emotions;
- Significant regional and seasonal differences in the impact of temperature on negative emotions, indicating that the moderating role of climate on mental health is highly dependent on interactions between regional climatic characteristics, seasonal patterns, and temperature metrics. Further validation integrating geographic climate data and social behavioral factors is needed.

However, this study has certain limitations, such as not comprehensively covering all meteorological factors and potential biases associated with reliance on search engine data. Future research should aim to integrate a wider variety of meteorological data and utilize multi-source data (e.g., social media, medical records) for a more comprehensive assessment of the impact of climate change on mental health. Additionally, given the variability in individual sensitivity, conducting personalized studies on emotional response patterns is also deemed necessary. Through these efforts, we can better understand and address the mental health challenges posed by climate change.

## Data Availability

The original contributions presented in the study are included in the article/Supplementary material, further inquiries can be directed to the corresponding author.

## Footnotes

^1^ https://index.baidu.com
